# Clinical assessment of tibial torsion differences. Do we always need a computed tomography?

**DOI:** 10.1007/s00068-022-01884-4

**Published:** 2022-02-10

**Authors:** Humam Hawi, Till Frederik Kaireit, Christian Krettek, Emmanouil Liodakis

**Affiliations:** 1 Trauma Department of the Hannover Medical School (MHH), Carl-Neuberg-Str. 1, 30625 Hannover, Germany; 2 Department of Diagnostic and Interventional Radiology, Hannover Medical School (MHH), Carl-Neuberg-Str. 1, 30625 Hannover, Germany

**Keywords:** Clinical exam, Tibial torsion, Reliability, Computed tomography

## Abstract

**Background:**

Tibial torsional malalignment presents a well-known complication of intramedullary nailing for tibial shaft fractures.

**Purpose:**

Objective of this study was to investigate the ability to clinically assess tibial torsion differences. Computed Tomography (CT) was used here as the gold standard. Further, intra- and inter-observer reliability of the clinical examination, and radiological measurements were calculated.

**Methods:**

Fifty-one patients with torsion-difference CTs, obtained for various reasons, were asked to kneel on an examination couch with free hanging feet. All patients are positioned with 90° flexed knee and neutral ankle. A picture of the lower extremities was obtained from the back of the patient. Two blinded orthopedic surgeons were asked to look at the pictures and measure the tibial torsion with a digital goniometer, based on the axis of the femur in relation to the second ray of the foot. To determine the intra-observer variation, the torsional angles were calculated again after 4 weeks. To be able to compare values, two blinded radiologists calculated torsional differences based on computed tomography.

**Results:**

All patients were able to be positioned for clinical assessment (*n* = 51). Clinical assessment of torsional difference revealed 4.55° ± 6.85 for the first, respectively, 4.55° ± 7.41 for the second investigator. The second measurement of the first investigator revealed a value of 4.57° ± 6.9. There was a good intra-observer agreement for clinical assessment (ICC 0.993, *p* < 0.001). Also, the inter-observer agreement showed a good inter-observer agreement (ICC 0.949, *p* < 0.001). Evaluation of radiological inter-observer assessment could also show a good inter-observer agreement (ICC 0.922, *p* < 0.001). The clinical method showed a good correlation to the CT method (0.839, *p* < 0.001). Additionally, the Bland–Altman plot was used to compare graphically both measurement techniques, which proved the agreement.

**Conclusion:**

In summary, computed tomography-assisted measurement of tibial torsion and clinical assessment correlated significantly good. In addition to that, clinical measurement has a good intra- and inter-observer reliability. Clinical examination is a reliable and cost-effective tool to detect mal-torsion and should be part of the repertoire of every surgeon.

## Introduction

Intramedullary nailing presents the most common treatment option for most tibia fractures. It is a reproducible and minimally invasive technique that offers rapid recovery for patients [1–5]. Despite these advantages, closed intramedullary nailing has been associated with high rates of torsional malalignment compared to open techniques [6–9].

Measurement of tibial torsion is done along the longitudinal axis of the tibia and is measured in comparison to the uninjured side. A discrepancy of more than 10° is defined according to most authors as malrotation [2–5, 9–11]. Currently, computed tomography is accepted as gold standard procedure for determining tibial torsion [2, 9, 12–16].

Tibial torsional malalignment presents a frequent and severe complication of intramedullary nailing for tibial shaft fractures. Post-interventional torsional malalignment of the tibia ranges according to several authors using computed tomography between 19 and 41% [1–4, 9–11, 17]. Recent published work underline the high risk of underestimation of torsional malalignment of the tibia. So, Puloski et al. describe an incidence of 22%, Cain et al. an incidence 36% and Theriault et al. even an incidence of 41% [4, 9, 11].

Torsional malalignment of the tibia has according to several reports effect on the clinical outcome as well ultimately financial impact [9, 18–22]. Therefore, early detection of malrotation of the tibia is of urgent importance.

Computed tomography (CT) is believed to provide the most accurate measurements and is therefore considered to be currently the gold standard for analyzing torsional alignment. Nonetheless, the associated radiation exposure and additional costs should not be underestimated [23]. Magnetic resonance imaging (MRI) represents a reliable radiation-free alternative [24]. However, MRI is expensive, time-consuming and susceptible to artifacts in the presence of metal implants, which is very common in orthopedic patients.

Contrary to the clinical evaluation of femoral antetorsion, which is complicated because of the ability of the hip joint to rotate, tibial torsion seems to be simpler to measure clinically [25]. The tibia is practically fixed between the knee and ankle joint, which have only minimal degrees of rotational freedom. In theory, this means that the foot can probably be reliably used as an indicator of tibial torsion if the knee joint and patella are positioned in a reproducible way, same for both lower limbs. Nonetheless, reports of clinical evaluation of tibial torsion seem to underestimate malalignment, given between 0 and 7%, which relies on the different measurement techniques and not reliable clinical assessments [2, 4]. Despite this, only a few studies have evaluated clinical examination as a measuring tool for tibial torsion [26].

Objective of this study was to investigate the ability to clinically assess tibial torsion differences. Computed Tomography (CT) was used here as the gold standard. Further, intra- and inter-observer reliability of the clinical examination, and radiological measurements were calculated.

## Patients and methods

Ethical board approval of this study was obtained (Nr. 8055_BO_K_2018) according the Declaration of Helsinki. Both postoperative rotational CT and clinical examination are part of the hospital’s protocol in case of suspected torsional malalignment.

### Design of the study

A prospective study was initiated at our institution (Level I trauma center) to evaluate the value of clinical examination to determine tibial torsional alignment. Patients, scheduled for postoperative follow-up either after treatment in our hospital or after referral from other institutions after fracture treatment or post-traumatic deformity, were included. Patients, who were not able to get mobilized for the purpose to kneel on their knee, patients with bilateral fractures, or intraarticular fractures were excluded. Fifty-one patients with lower limb long bone fractures and a suspected torsional malalignment got a low-dose torsional difference CT. Additionally, tibial torsion was clinically assessed according to the below mentioned procedure. The time from operation to torsional measurement (CT and clinical) was 1–12 weeks for all patients. Although participation in the study was not mandatory, no patient denied participating to the study.

### Patients

Fifty-one patients with a postoperative CT scan for torsional assessment were included. Thirty-nine of the patients were male and 12 female. The mean age was 47 ± 18.5 (range 16–79) years.

### CT- determination of tibial torsion

Scans were obtained with the LightSpeed QX/i CT equipment (GE Healthcare, USA) while the lower limbs were extended and mounted to a foot-rest to stabilize their position during scans. Sections of 1.25 mm thickness were taken through the hip, knee and ankle joints.

**Fig. 1:**
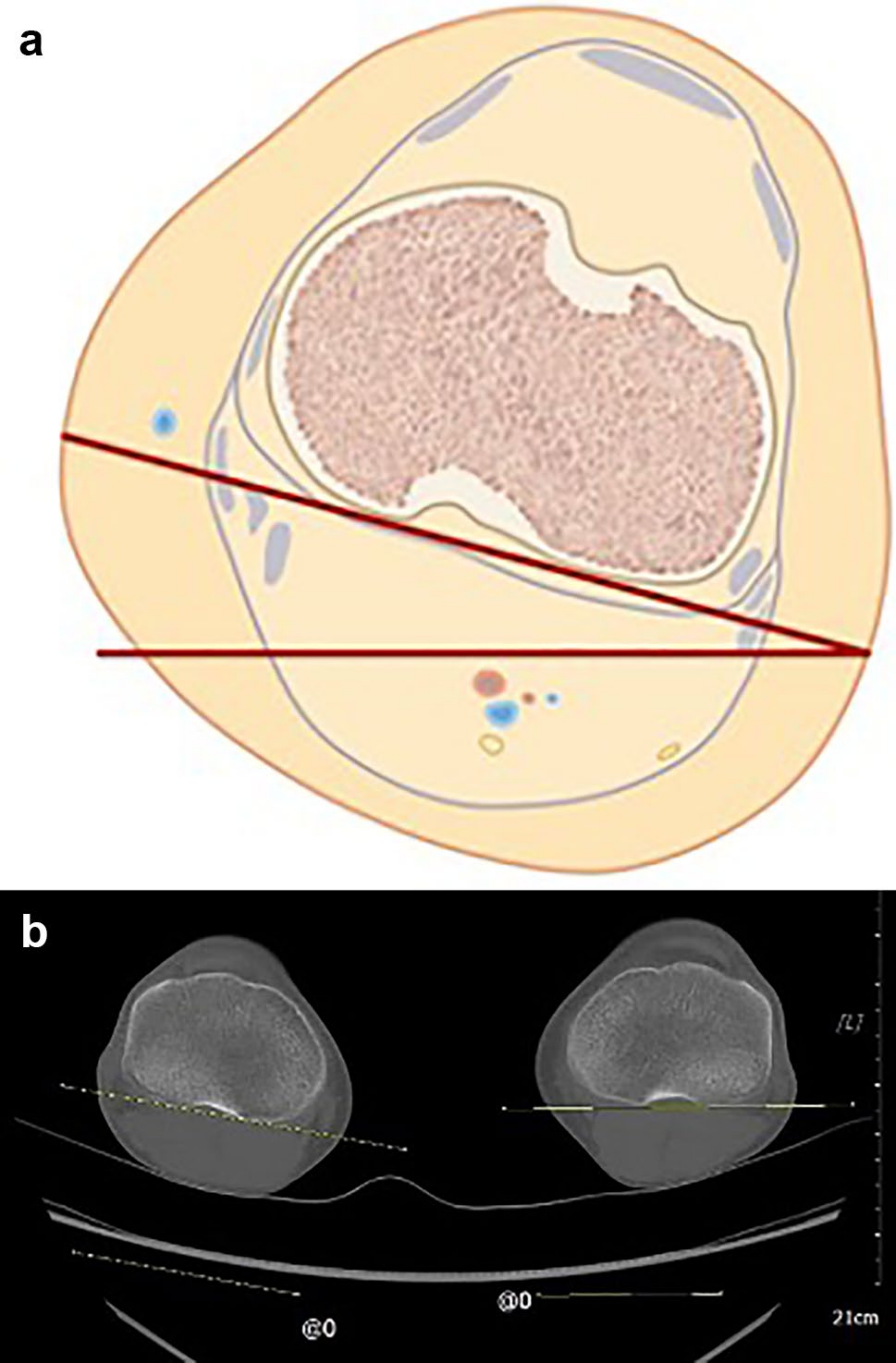
**a** Illustration of measuring the proximal tibial axis. **b** Measurement of the proximal tibial axis using CT

**Fig. 2:**
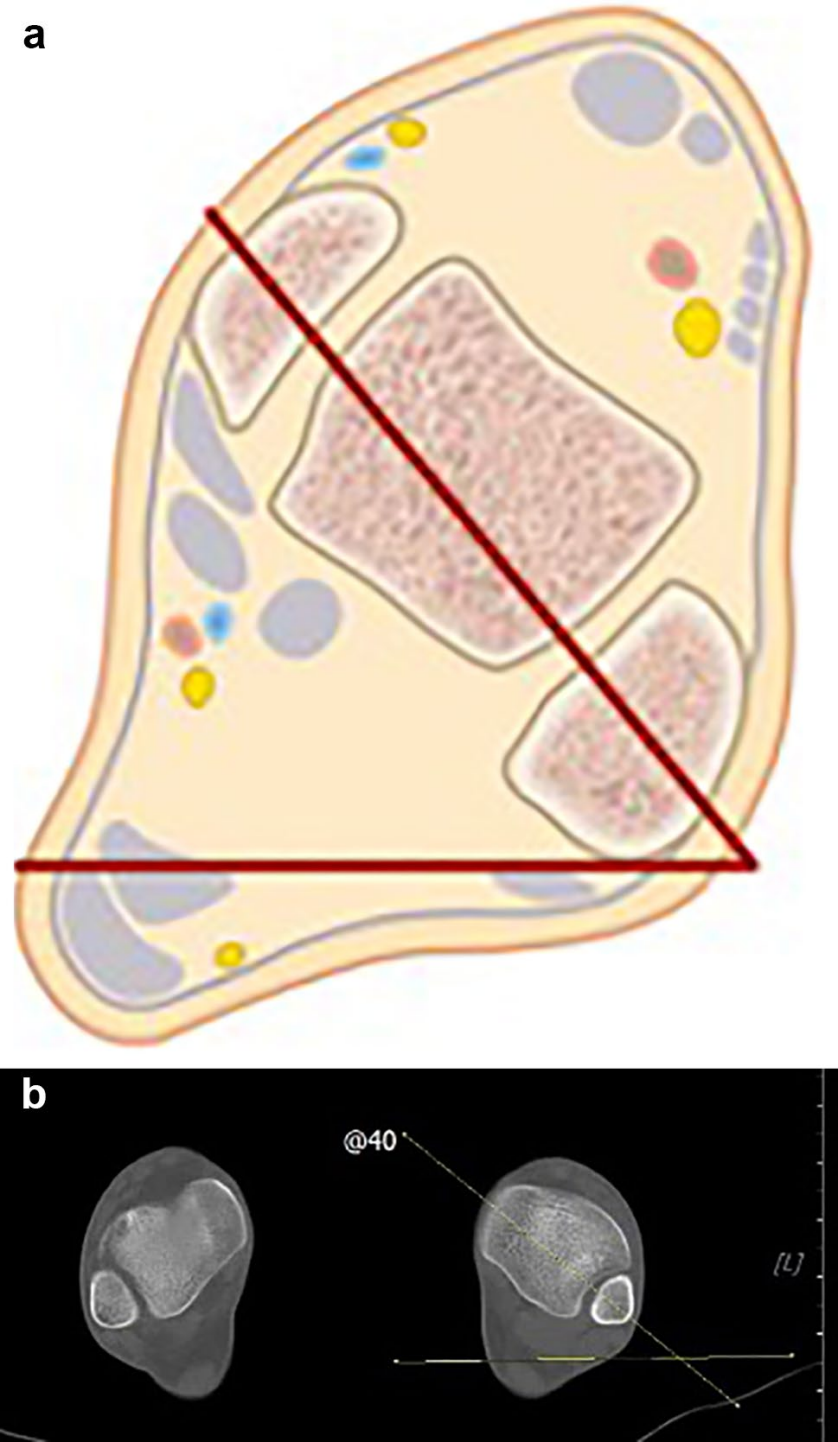
**a** Illustration of measuring the distal tibial axis. **b** Measurement of the distal tibial axis using CT. Torsional alignment measurement reveals a difference of 12° in comparison to the contralateral side

To analyze the tibial torsional profile, two angles where measured:

1. the angle between the proximal tibial axis (dorsal tangent to the tibial plateau) and a horizontal line (proximal tibial angle) (Fig. 1),
2. the angle between the bi-malleolar axis and a horizontal line (distal tibial angle) (Fig. 2).

The proximal tibial axis is defined as the dorsal tangent to the tibial plateau, whereas as distal tibial axis the line connecting the centers of the dense surfaces of the malleoli is chosen (Fig. 2) [27]. Tibial torsion was calculated by subtracting the proximal tibial angle from the distal tibial angle. This method of measuring tibial torsion has been chosen over other methods because of the high intra- and inter-observer reliability shown in recent studies [14].

Data analysis was done with the use of FDA approved medical planning software (MediCAD version 2.0, Hectec GmbH, Altfraunhofen, Germany). Internal torsion was assigned a minus (−) sign and external torsion a positive (+) sign. Torsional alignment of the tibia was measured by two experienced radiologists for inter-observer reliability.

### Clinical examination

For clinical examination purposes, the patients were asked to kneel on an examination couch with free hanging feet. Patients were positioned with 90° flexed knee and neutral ankle. Additionally, the hips were positioned in neutral abduction/adduction and the back straight.

**Fig. 3:**
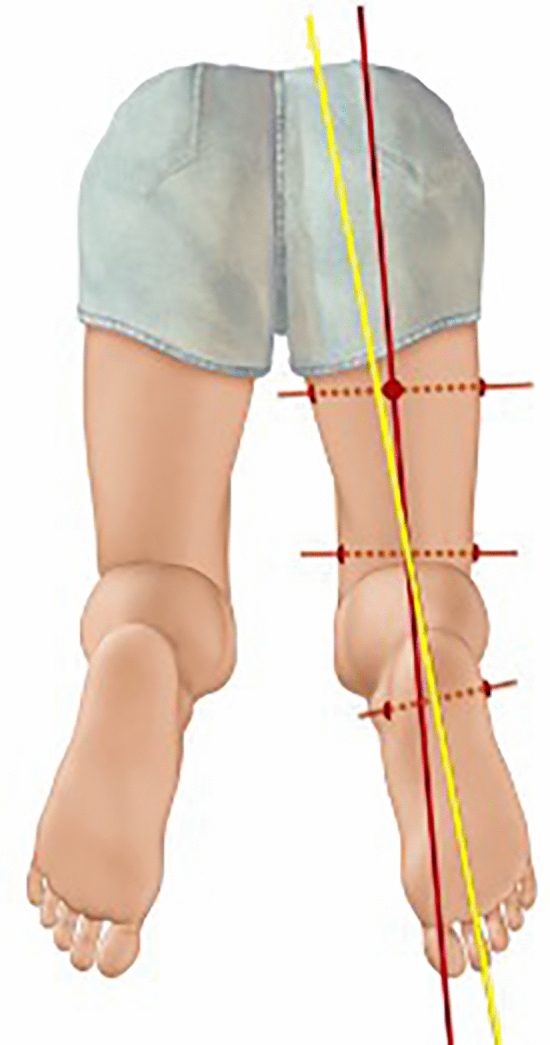
Illustration of clinical assessment of tibial torsion with the thigh–foot angle (TFA) (red line) and the second metatarsal axis (yellow line)

**Fig. 4:**
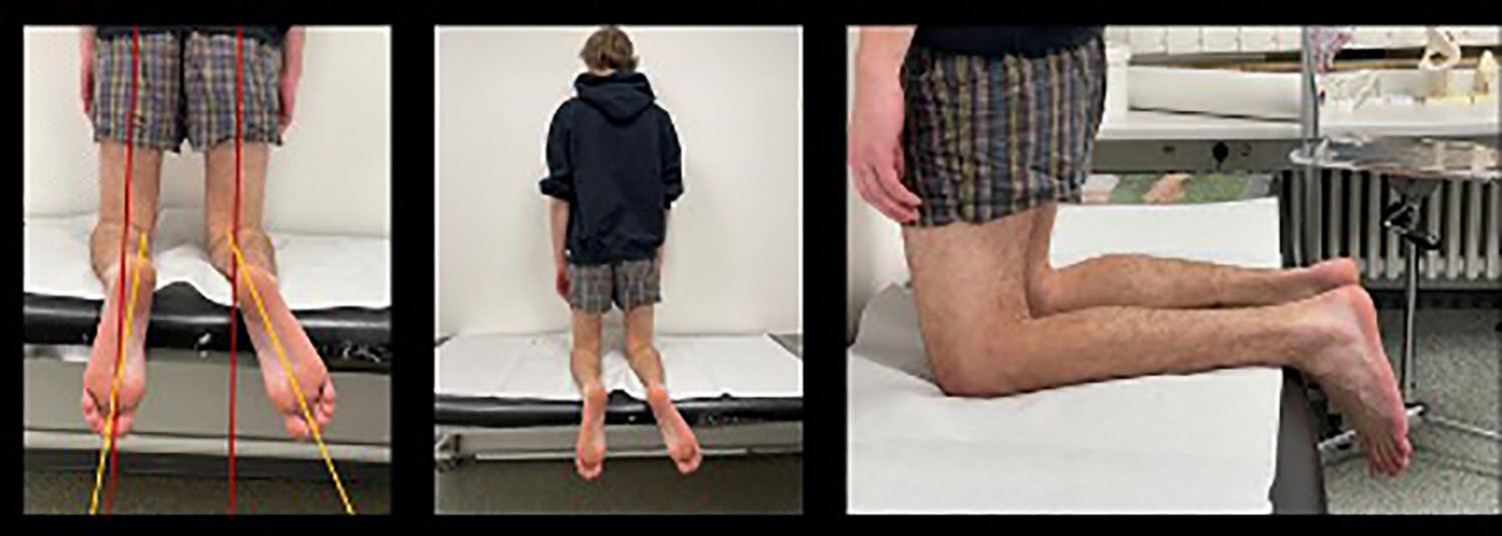
Example of clinical examination using the above-mentioned measuring technique revealing a torsion difference of 15°. Same patient as in Figs. 1 and 2

A digital picture, strictly parallel to the sole of the feet and depicting both thighs and both feet, is obtained. Torsion is measured using a digital goniometer with the axes of the femur and the second ray of the feet. Differences are calculated in comparison to the contralateral side. (Figs. 3, 4) The pictures were imported into a personal computer and a line along the thigh axis and the second metatarsal axis was drawn. The angle between these two lines represented the tibial torsion angle.

Data analysis was done digitally with the use of FDA approved medical planning software (MediCAD version 2.0, Hectec GmbH, Altfraunhofen, Germany). Internal torsion was assigned a minus (-) sign and external torsion a positive (+) sign. Measurements were performed by two experienced orthopaedic trauma surgeons, one surgeon measured again after 4 weeks to look for both inter- and intra-observer variation.

### Statistical analysis

Continuous variables were checked for normal distribution using the Shapiro–Wilk test, and presented in the form of mean ± SD (standard deviation). Statistical significance was evaluated between different groups using the paired t test. Intra-observer and inter-observer reliability was evaluated using the intra-class correlation coefficient (ICC). The two-way mixed model (absolute agreement) was used. The scoring system of Fleiss et al. [28] was utilized in the analysis of our results (good > 0.75, fair 0.4–0.75, poor < 0.4). A *p* value ≤ 0.05 (two-tailed) was considered to be statistically significant. We used the Bland–Altman plot to compare graphically both measurement techniques [29–31]. All statistical analyses were performed using SPSS (SPSS 23.0, SPSS Inc., Chicago, IL, USA).

## Results (Tables 1, 2)

**Table 1:** Assessment of the Thigh–Foot Angle and the radiological assessment

		Value (°)	SD (°)	Correlation	
		Thigh-Foot Angle (TFA)			
Observer 1	Measurement 1	4.57	6.90	ICC 0.993 *p* < 0.001	
	Measurement 2	4.55	6.85		ICC 0.949 *p* < 0.001
Observer 2		4.55	7.41		
		CT			
Observer 1		3.20	9.06	ICC 0.922 *p* < 0.001	
Observer 2		4.10	8.36		

**Table 2:** Mean difference and comparison of torsional difference between both methods

	Value (°)	SD (°)	Correlation	Mean Difference (°)
	Thigh-Foot Angle (TFA)		ICC 0.839 *p* < 0.001	− 0.451 ± 4.55 *p* > 0.05
Observer 1	4.55	6.85		
	CT			
Observer 2	4.10	8.36		

The complete data are summarized in Tables 1 and 2.

### Thigh–Foot Angle measurement of torsional difference

Clinical assessment of torsional difference revealed a value of 4.55° ± 6.85 by the first investigator and 4.57° ± 6.90 in the second series. Measurements by the second investigator revealed a value of 4.55° ± 7.41. There was a good intra-observer agreement between both measurements with ICC 0.993 (*p* < 0.001). Comparison between investigator one in the first measurement series and investigator two revealed a good inter-observer agreement with ICC 0.949 (*p* < 0.001).

### Radiological measurement of torsional difference

The measurements by computed tomography revealed a value of 3.20° ± 9.06 for the first investigator and 4.10° ± 8.36° for the second investigator. There was a good inter-observer agreement with ICC 0.922 (*p* < 0.001).

### Mean differences of absolute torsion values by both methods

Mean differences of absolute torsion measurements revealed a value of 17.22° ± 10.53 (*p* < 0.001) for the right side (ICC = 0.160, *p* > 0.05) and 17.57° ± 11.11 (*p* < 0.001) for the left side (ICC = 0.138, *p* > 0.05).

### Comparison of torsional difference measurements by both methods

Comparison of clinical and radiological assessment revealed a good correlation with 0.839 (*p* < 0.001). Mean difference between both methods revealed a value of − 0.45 ± 4.55 (*p* > 0.05).

**Fig. 5:**
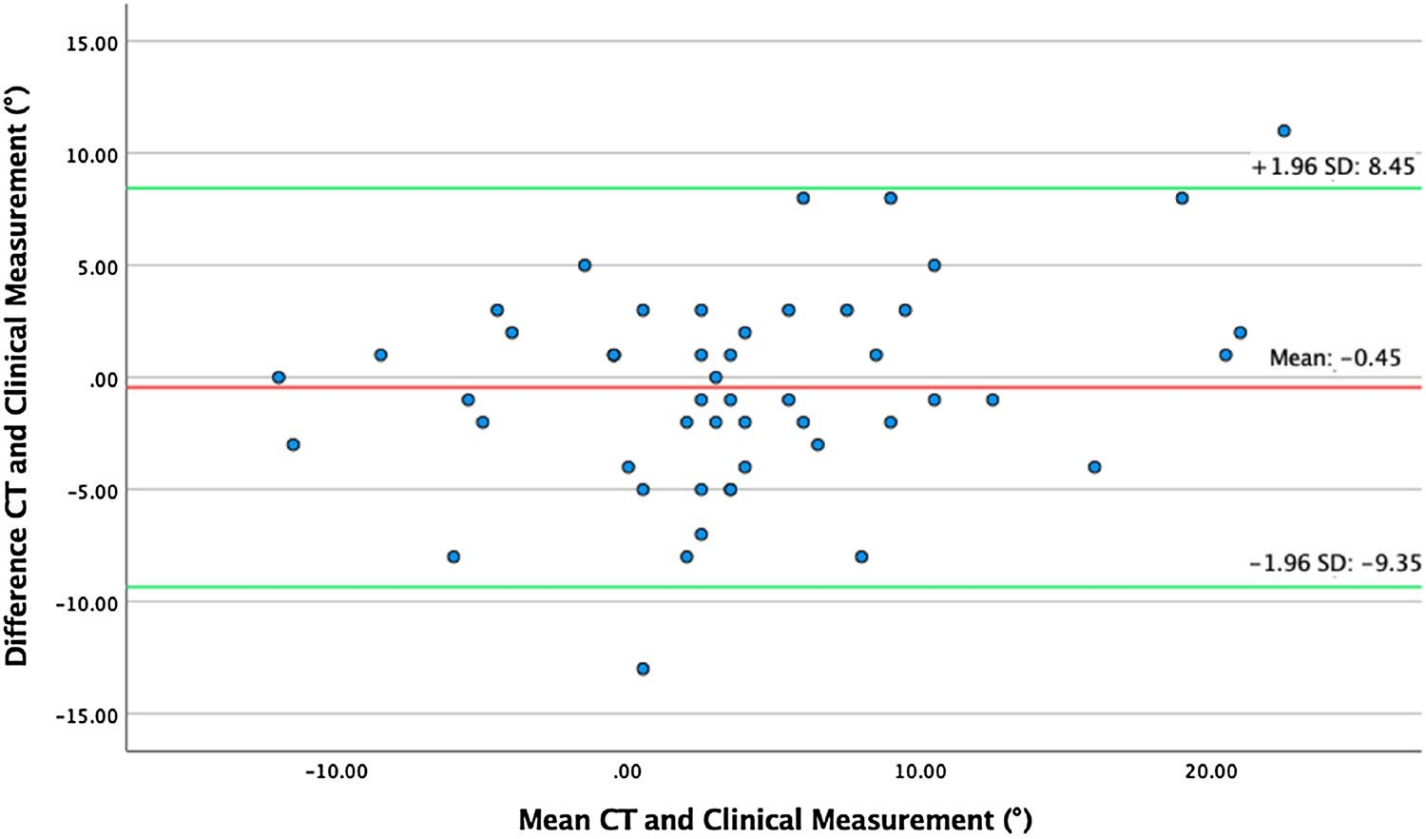
Analysis of both measurement techniques by the Bland–Altman plot visualized the agreement

Analysis of both measurement techniques by the Bland–Altman plot visualized the agreement (Fig. 5). Horizontal lines are drawn at the mean difference. The limits of agreement are defined as the mean difference plus and minus 1.96 times of the standard deviation of the differences. We could show, that these limits do not exceed the maximum allowed difference between measurement methods, except in two cases where the allowed difference is slightly exceeded. Careful interpretation of values is considered, that there is an agreement between both techniques and that techniques may be used interchangeably.

## Discussion

The accuracy of a test can be defined as how close a measured value is to a true value. In this case it means, how close the clinical measurement of tibial torsion is to the real torsion measured by CT or in a cadaver. A highly accurate method should also be reliable when used by the same observer for repeated measurements (intra-observer reliability) or even by different observers (inter-observer reliability). Our results showed that clinical measurements of tibial torsion differences are highly accurate and have a great inter- and intra-observer reliability.

Intraoperative tools for tibial torsion control are limited. The cortical step sign and the diameter difference sign have been described as potential tools to identify torsional malalignment intraoperatively. Keppler et al. evaluated in a recent cadaveric study the cortical step sign and the diameter difference sign in mid-shaft fractures of the tibia [32]. They came to the conclusion that torsional discrepancies in tibial mid-shaft fractures can be most reliably assessed in the lateral plane by analysis of the lateral cortical thickness and tibial diameter.

CT is widely accepted as the most accurate method for analyzing torsional malalignment. A previous study from our research group showed that the inter-observer reliability of CT measurements using the bi-malleolar method is 0.92 and the intra-observer reliability between 0.996 and 0.999 [14]. Compared to that, the inter- and intra-observer reliabilities of the clinical examination of tibial torsional differences are slightly lower but still very good (ICC = 0.949 for interoberver and ICC = 0.993 for intraobserver reliability). The main advantage of using the clinical exam instead of CT is that the radiation associated with a CT for torsional profile (between 0.3 and 0.5 mSv per scan) can be avoided [23, 24, 33]. Taken into consideration that often pediatric patients are suspected for tibial malrotation, radiation exposure is a relevant issue.

Sestan et al. [34] developed and evaluated a new Torsiometer to facilitate non-invasively and radiation-free measurements of the tibial torsion. This device consists of a freely rotating telescoping tube and rubberized malleollar and epicondylar cups. The authors compared the measurements acquired by the Torsiometer with CT measurements and report very accurate and reliable results. Despite the reported good results, we believe that palpating the femoral epicondyles is often inaccurate. This is why knee arthroplasty surgeons do not rely solely on the trans-epicondylar axis but also use the posterior condylar and the anteroposterior axis [35]. Τo the best of our knowledge, the device has not been widely accepted by the orthopedic community and there are no further studies confirming these results.

Tibial torsion can be evaluated in various different positions (prone, supine, seating etc.). King and Staheli described 1984 a goniometrical method to record tibiofibular torsion and Thigh–Foot Angle. The positioning of the subjects was prone and the knee flexed to 90°, and the ankle positioned in neutral dorsi-flexion/plantar-flexion. The authors paid attention for relaxation of the subjects’ leg to eliminate the influence of the hamstring muscles [36]. Stuberg et al. compared this measurement method already to computed tomography. [26] The authors conclude, that clinical measurement of tibial torsion is a reproducible measurement method within an acceptable range.

Jakob et al. described in 1981 the measurement of the thigh foot angle in a seating position [37], whereas Bouchard et al. measured in 2004 the angle between the foot axis and the examination stretcher in patients lying horizontally on the stretcher (supine position) [38]. The main advantage of all above described variations is the patient comfort during the examination (especially in patients treated with an intramedullary tibial nail). However, the disadvantage is the lack of standardization of the clinical exam. By placing the patient in a kneeling position, the hip, knee and foot position is fixed in a reproducible position without the need of the patient actively holding the leg and without the need of an examiner supporting the leg. Additionally using the kneeling method, standardization of observer position can be easily achieved by taking the digital picture strictly from posterior. Computer-assisted analysis of the picture offers the advantage of drawing the axes more accurately. This leads to a better positioning of the arms and fulcrum of the goniometer and finally to a more accurate measurement. The picture with the measurement can also be digitally stored, which contributes to a better documentation.

The study has some limitations. Kneeling can be difficult in the early postoperative period, especially after tibial nailing, because of anterior knee pain. Despite the fact that this is the biggest study comparing clinically measured tibial torsion with CT, the sample size was relatively small.

In conclusion, our null hypothesis that there is no difference between clinical and CT measurements of tibial torsion, could not be rejected. We also showed that the clinical exam, when performed as described above, has a high inter- and intra-observer reliability, comparable to that of CT. Despite these results, CT is a highly accurate method for measuring torsional deformities and we still recommend the use of CT before performing revision surgery to correct tibial torsion malalignment.
